# Neuron-Glia Crosstalk Plays a Major Role in the Neurotoxic Effects of Ketamine *via* Extracellular Vesicles

**DOI:** 10.3389/fcell.2021.691648

**Published:** 2021-09-16

**Authors:** Donald H. Penning, Simona Cazacu, Aharon Brodie, Vesna Jevtovic-Todorovic, Steve N. Kalkanis, Michael Lewis, Chaya Brodie

**Affiliations:** ^1^Department of Anesthesiology, Pain Management and Perioperative Medicine, Henry Ford Hospital, Detroit, MI, United States; ^2^Department of Neurosurgery, Henry Ford Health System, Detroit, MI, United States; ^3^Precise Cell Ltd., Petach Tikva, Israel; ^4^Department of Anesthesiology, University of Colorado School of Medicine, Aurora, CO, United States; ^5^Faculty of Life Sciences, Bar-Ilan University, Ramat Gan, Israel

**Keywords:** ketamine, BDNF, BDNF-AS, neurotoxicity, astrocytes, microglia

## Abstract

**Background:** There is a compelling evidence from animal models that early exposure to clinically relevant general anesthetics (GAs) interferes with brain development, resulting in long-lasting cognitive impairments. Human studies have been inconclusive and are challenging due to numerous confounding factors. Here, we employed primary human neural cells to analyze ketamine neurotoxic effects focusing on the role of glial cells and their activation state. We also explored the roles of astrocyte-derived extracellular vesicles (EVs) and different components of the brain-derived neurotrophic factor (BDNF) pathway.

**Methods:** Ketamine effects on cell death were analyzed using live/dead assay, caspase 3 activity and PARP-1 cleavage. Astrocytic and microglial cell differentiation was determined using RT-PCR, ELISA and phagocytosis assay. The impact of the neuron-glial cell interactions in the neurotoxic effects of ketamine was analyzed using transwell cultures. In addition, the role of isolated and secreted EVs in this cross-talk were studied. The expression and function of different components of the BDNF pathway were analyzed using ELISA, RT-PCR and gene silencing.

**Results:** Ketamine induced neuronal and oligodendrocytic cell apoptosis and promoted pro-inflammatory astrocyte (A1) and microglia (M1) phenotypes. Astrocytes and microglia enhanced the neurotoxic effects of ketamine on neuronal cells, whereas neurons increased oligodendrocyte cell death. Ketamine modulated different components in the BDNF pathway: decreasing BDNF secretion in neurons and astrocytes while increasing the expression of p75 in neurons and that of BDNF-AS and pro-BDNF secretion in both neurons and astrocytes. We demonstrated an important role of EVs secreted by ketamine-treated astrocytes in neuronal cell death and a role for EV-associated BDNF-AS in this effect.

**Conclusions:** Ketamine exerted a neurotoxic effect on neural cells by impacting both neuronal and non-neuronal cells. The BDNF pathway and astrocyte-derived EVs represent important mediators of ketamine effects. These results contribute to a better understanding of ketamine neurotoxic effects in humans and to the development of potential approaches to decrease its neurodevelopmental impact.

## Introduction

Prolonged and multiple exposure of general anesthetics (GAs) have been reported to impair the development of the immature brain by inducing neurotoxicity and impacting the cerebral cytoarchitecture (Jevtovic-Todorovic et al., 2003; Jevtovic-Todorovic, 2016, 2018). Most studies were mainly conducted in rodents and non-human primates. Far fewer studies have been conducted in humans, have focused mainly on cognitive and behavioral changes, and are not conclusive (Davidson et al., 2016; Sun et al., 2016; McCann and Soriano, 2019; McCann et al., 2019). Studies of developmental neurotoxicity of anesthetics in humans are challenging, behavioral assessments are difficult and must account for other confounding factors. These include inability to dissociate the effects of anesthetics from the surgical process, underlying pathologies for which the surgery was required, and the stress of illness and surgery. These factors are especially important when extrapolating studies from animals to humans, which have their brain growth spurt at different times (Dobbing and Sands, 1979). In addition, recent reports have highlighted the differences in the functions of various cells in the central nervous system (CNS) in humans and rodents (Akter et al., 2020). Therefore, there is a need to analyze GAs neurotoxic effects and mechanisms in reliable human models to supplement pre-clinical animal studies.

There is now powerful evidence for the role of non-cell autonomous mechanisms in various pathological conditions in the CNS which shows that neurotoxicity is strongly affected by changes in both neuronal and non-neuronal cells (Ilieva et al., 2009; Meyer et al., 2014; Kim et al., 2016; Zanghi and Jevtovic-Todorovic, 2017; Zhang et al., 2019; Zhou et al., 2019). These mechanisms are especially relevant to glial cells, including astrocytes, oligodendrocytes and microglia, all of which exhibit structural and functional interactions with neurons. Although some studies have demonstrated a role of astrocytes (Zhang et al., 2019; Zhou et al., 2019) and microglia (Baud and Saint-Faust, 2019) in GA effects, the non-cell autonomous effects of different human glial cells in this process have not been consistently identified and studied.

Extracellular vesicles (EVs) have been recently identified as a major mechanism for intercellular communication in the CNS (Basso and Bonetto, 2016). These vesicles carry a specific cargo consisting of RNA molecules, proteins and lipids which play important roles in both physiological and pathological pathways. In the CNS, EVs play a critical role in neuron-glia interactions (Datta Chaudhuri et al., 2020) and dysregulated EV-related communication has been implicated in a variety of pathological conditions, including stroke, brain injury and neurodevelopmental disorders. However, the role of EVs in the neuron-glia crosstalk during GA-induced neurotoxicity has not been yet defined.

While gamma-amino-butyric acid type-A receptors (GABAAR) (Weir et al., 2017) and *N*-methyl-D-aspartate receptors (NMDAR) (Sachana et al., 2018) represent major factors in GA-induced neurotoxicity, other mechanisms related to cell apoptosis and autophagy, calcium concentrations and epigenetic modifications have been also implicated (Lei et al., 2012; Twaroski et al., 2015; Yu et al., 2017). In addition, the regulation of neurotrophin expression, in particular brain-derived neurotrophic factor (BDNF), has been implicated in a variety of pathological conditions in the CNS (Fukumoto et al., 2019) including GA-induced neurotoxicity (Lu et al., 2006; Ozer et al., 2015).

This study is based on the hypothesis that the non-cell autonomous interaction of glial and neuronal cells plays an important role in ketamine-induced neurotoxicity and that this interaction is mediated, at least in part, *via* extracellular vesicles. Here, we analyzed the effects of ketamine on the functions of human neural cells and the cellular and molecular mechanisms that mediate these effects. We found that ketamine induced cell death in neurons and oligodendrocytes and promoted the activation of astrocytes and microglia toward the pro-inflammatory A1 and M1 phenotypes, respectively. We demonstrated an enhanced neurotoxic effect of ketamine in neuron-glia co-cultures indicating an important contribution of both cell and non-cell autonomous mechanisms. Finally, we identified the BDNF/pro-BDNF/BDNF-AS pathway as a major pathway and extracellular vesicles as potential mediators of ketamine neurotoxicity.

## Materials and Methods

### Materials

Human BDNF and Active Pro-BDNF ELISA Kits (DEIA-XY2236) were obtained from Creative Diagnostics (Shirley, NY, United States). Human IL-13 (ab178014) and IL-1β (ab214025) ELISA kits, were obtained from Abcam (Cambridge, MA, United States).

### Neural Cell Cultures

Human hTERT immortalized human fetal microglial cells (Bier et al., 2020) and astrocytes were obtained from Applied Biological Material (Richmond, BC, Canada). Human neurons and oligodendrocyte precursor cells were obtained from ScienCell (Carlsbad, CA, United States). The different cells were maintained in growth media and conditions recommended by the manufacturers. All cells employed in this study were tested for mycoplasma contamination (Mycoplasma PCR Detection Kit) and found negative.

### Experimental Protocols

Cell cultures were treated with different ketamine concentrations for 6 h, the culture medium was changed and cells were then analyzed following 24–48 h as indicated in the specific experiments. Co-cultures of neurons and glial cells were maintained in neuronal cell medium (ScienCell) and in these experiments, individual glial cultures were maintained in the same medium.

All experiments were performed in medium containing EV-depleted serum. EV-depleted FBS was prepared by overnight centrifugation (100,000 × *g*, 4°C) followed by filtration of the supernatant in a 0.22 μm filter.

Treatment of astrocytes with GW4869 to inhibit EV secretion and silencing of BDNA-AS in these cells were performed prior to their co-culture with neurons.

### Microglia and Astrocyte Activation State

Human microglia cells and astrocytes were analyzed for the expression of M1 and M2 or A1 and A2 markers, respectively using real-time-PCR, Western blot analysis and ELISA.

### Cell Growth and Proliferation

Cells were plated at a concentration of 2,000 cells/well in 96 wells. Cell proliferation was determined using the ViaLight plus kit (LT07-221, Lonza, Walkersville, MD, United States) according to the manufacturer’s guidelines.

### Cytotoxic Assays

#### LDH Assay

Cells were treated with different concentrations of ketamine. Following treatment, the cells were analyzed for cell death using LDH assay (MAK066-1KT, Sigma-Aldrich (St. Louis, MO, United States).

#### Live/Dead Assay

The live/dead cell assay (MP03224, Molecular Probes, Invitrogen) was performed as described previously (Jiang et al., 2016). Briefly, calcein AM and EthD-1 were added to the culture medium at a final concentration of 1 μM for calcein AM and 2.5 μM for EthD-1 and the relative live/dead cell number was then analyzed.

### Western Blot Analysis

Western blot analysis was performed as previously reported (Jiang et al., 2016; Bier et al., 2018). Briefly, cell lysates were solubilized with RIPA buffer supplemented with PhosSTOP and Complete Phosphatase/Protease Inhibitor Cocktails (Roche Diagnostics) and protein content was analyzed using a standard BCA assay. Protein extract (20–30 μg per sample) were loaded on sodium dodecyl sulfate-polyacrylamide electrophoresis gels and transferred to PVDF membranes that were probed with the specific antibodies as detailed. Bound antibodies were visualized with an enhanced chemiluminescence detection kit (Amersham Pharma-Biotech). Equal loading was verified using an anti actin antibody. The following primary antibodies were used: Anti-TrkB (Cat# sc-136990, 1:500), anti-p75 (Cat# sc-13577, 1:1,000), and anti-EAAT2 antibodies (Cat# sc-365634, 1:500) (Santa Cruz Biotechnology). Anti-cleaved PARP1 (AB3820, 1:1,000), Anti-cleaved caspase 3 (ab2303, 1:500), anti-C3 (ab97462, 1:500) anti-S100A10 (ab76472, 1:500) and exosome panel antibody (ab275018) (Abcam, Cambridge, MA United States). Anti-mouse and anti-rabbit HRP secondary antibodies (1:10,000, Pierce).

#### Caspase 3 Activity

Caspase 3 activity was performed using a fluorometric assay according to the manufacturer’s instructions (abcam). The data were calculated as fluorescence units/mg protein and presented as fold increase over the control level.

### Quantification of Cytokine Secretion

Supernatants were collected and stored at −80°C. Pro-BDNF, BDNF, IL-1, and IL-13 secretion were quantified using enzyme-linked immunosorbent assays (ELISA) according to the manufacturer’s instructions. The concentrations of the specific cytokines were calculated using a standard curve and were expressed as picograms per milliliter.

### Real-Time PCR

Total RNA was extracted using RNeasy mini kit according to the manufacturer’s instructions (Qiagen, Frederick, MD, United States). Reverse transcription reaction was carried out using 2-μg total RNA as previously described (Morgoulis et al., 2019; Bier et al., 2020). Briefly, reactions were run on an ABI VIIA7 Sequence Detection System (Applied Biosystems, Foster City, CA, United States). Cycle threshold (Ct) values were obtained from the ABI QuantStudio software. S12 ribosomal protein levels were used as controls. The primer sequences are described in Supplementary Table 1.

### Phagocytosis Analysis

Human microglial cells were treated with ketamine and phagocytosis was determined using the pHrodo^TM^ Green zymosan bioparticle assay (Invitrogen, Carlsbad, CA, United States) according to the manufacturer’s instructions. Briefly, microglia were incubated with a solution of pHrodo Green zymosan bioparticles in Live Cell Imaging Solution (0.5 mg/ml) for 2 h and the level of phagocytosis was analyzed using fluorescence plate reader at Ex/Em 509/533.

### Preparation of Extracellular Vesicles

Isolation of EVs from culture supernatants was performed using the ExoQuick-TC Ultra kit (SBI, Palo Alto, CA, United States) according to the manufacturer’s instructions. The protein content of the isolated EVs was determined using the Micro BCA assay kit (ThermoFischer Scientific, Oregon City, OR, United States), and the EV markers CD63 and CD81 were analyzed by Western blot. The quantification of the isolated EVs was performed using the ExoELISA-Ultra CD63 and CD81 kits (SBI, Palo Alto, CA, United States) according to the manufacturer’s instructions and as recently described (Bier et al., 2020). For EV treatment, 2 × 10^8^ EVs were administered to the cultured cells.

### Nanoparticle Tracking Analysis

Concentration and size distribution of isolated EVs were analyzed using NanoSight LM10 equipped with sCMOS camera and 405 nm laser (Malvern Instruments, MA, United States). Briefly, samples were thawed to room temperature and diluted with PBS to a concentration of approximately 10^8^ particles/ml. The EVs were injected into sample chamber and analysis of 60 s for each sample was captured at 25°C and processed using NTA 3.3 software.

### Statistical Analysis

Cultures were viewed microscopically and randomly assigned to the different experimental groups. All data collection and analysis were performed blinded to treatment groups. Data are representative or presented as the mean values ± SD of three to six independent experiments. The statistical difference between two groups was determined using unpaired, two-tailed student’s *t*-test. For comparisons between multiple groups a one-way ANOVA with Bonferroni-corrected *post hoc* was performed. *P*-value of < 0.05 was considered significant.

## Results

### Ketamine Induces Cell Death of Cultured Neuronal Cells

**FIGURE 1:**
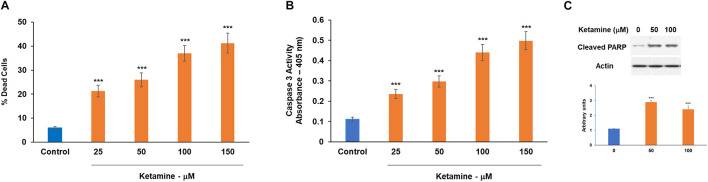
Neurotoxic effects of ketamine on human neuronal cells. Human neurons were treated with ketamine for 6 h. The medium was replaced with fresh medium and% dead cells was determined following 48 h using the live/dead assay **(A)**, caspase 3 activity **(B)** and analysis of cleaved PARP by Western blot analysis **(C)**. The results are the means ± SD of three different experiments analyzed in quadruplet **(A,B)** or a representative of three independent experiments **(C)** ***P < 0.001.

Ketamine has been reported to induce neurotoxicity in a variety of cellular and animal models. However, the effects and mechanisms of ketamine in human neural cells are just beginning to be understood. Here, we studied the effects of ketamine on human cultured neural cells focusing mainly on the role of the neuron-glial crosstalk. We first examined the neurotoxic effects of ketamine on human neuronal cultures. Based on multiple *in vitro* studies (Baker et al., 2016; Zhang et al., 2019; Kamp et al., 2020), we employed different ketamine concentrations (10–150 μM, Supplementary Figure 1A) and treatment time points (100 μM ketamine, 6 and 24 h, in neurons, Supplementary Figure 1B and oligodendrocyte progenitor cell, Supplementary Figure 1C. Human neuronal cultures exhibited a dose-dependent increased cell death in response to ketamine treatment (6 h, 25–150 μM) as assessed with dead/live assay (Figure 1A), increased caspase 3 activity (Figure 1B) and expression of cleaved PARP1 (Figure 1C). In view of the similarities in ketamine effects after 6 and 24 h, we used a 6 h ketamine treatment in the following experiments.

### Ketamine Induces a Relative Increase in A1 Astrocytic Phenotypes

Glial cells are implicated in the pathogenesis of multiple diseases and pathological conditions in the CNS. Astrocytes undergo activation into distinct reactive astrocyte subtypes in response to specific pathological conditions. Recently two main phenotypes of reactive astrocytes have been reported, namely, A1 and A2. It is currently accepted that there is an array of intermediate activation states that play major roles in various pathological conditions (Guttenplan et al., 2020). An increased A1 phenotype has been associated with neurodegenerative diseases and aging (Clarke et al., 2018; Yun et al., 2018; Miyamoto et al., 2020).

**FIGURE 2:**
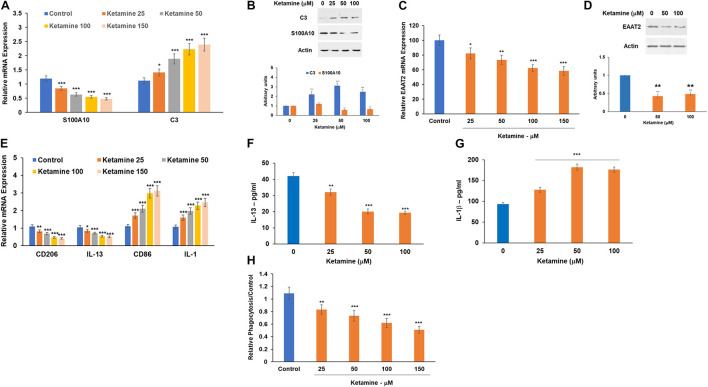
Ketamine induces activation of astrocytes and microglia cells. Human astrocytes were treated with ketamine and were analyzed after 48 h for the expression of A1 (C3) and the A2 (S100A10) markers using RT-PCR **(A)** and Western blot analysis **(B).** The expression of the glutamate transporter EAAT2 mRNA was analyzed using RT-PCR **(C)** and Western blot analysis **(D).** Human microglia cells were treated with ketamine and the relative expression of M1 and M2 markers was analyzed using RT-PCR **(E).** The ketamine treated microglia cells were also analyzed for secretion of IL-1β **(F)** and IL-13 **(G)** using ELISA and for phagocytosis using the pHrodo^TM^ assay **(H).** The results are the means ± SD of a three independent experiments **(A,C,E,H)** or are a representative of three independent experiments **(B,D,F,G)**. *P < 0.05, **P < 0.01, and ***P < 0.001.

We analyzed the effect of ketamine on the expression of the recently reported A1 (C3) and A2 (S100A10)-specific markers (Liddelow and Barres, 2017; Liddelow et al., 2017; Hinkle et al., 2019) using RT-PCR and Western blot analysis. Treatment with ketamine induced an upregulation of C3 mRNA (Figure 2A) and protein (Figure 2B) levels while decreasing the expression of S100A10 (Figures 2A,B), suggesting that this treatment promotes astrocyte activation toward the A1 phenotype. We also analyzed the expression of EAAT2 which mediates the uptake of glutamate by astrocytes and found that ketamine treatment decreased the mRNA (Figure 2C) and protein (Figure 2D) levels of this glutamate transporter.

In contrast to its effects on neurons, ketamine did not induce a significant effect on cell death in treated astrocytes (Supplementary Figure 2A).

### Ketamine Increases Microglia M1 Phenotypes

Microglia have been implicated in the pathogenesis of various diseases in the CNS *via* regulation of neuroinflammation, synapse function and glutamine homeostasis (Zhang et al., 2017; Clark et al., 2019). In addition, microglia activation and polarization into M1 subtype has been implicated in the activation of A1 astrocytes (Yun et al., 2018; Clark et al., 2019). Human microglia cells were treated with ketamine and the relative expression of commonly used M1 (IL-1 and CD86) and M2 (CD206 and IL-13) markers were determined using RT-PCR. Ketamine treatment induced a relative shift toward M1 polarization (Figure 2E). Similarly, treatment of astrocytes with ketamine decreased IL-13 (Figure 2F) and increased IL-1β (Figure 2G) secretion as determined by ELISA. We also found that ketamine treatment decreased microglia phagocytosis as demonstrated in Figure 2H. Similar to its effects in astrocytes, ketamine did not induce a significant cell death in the microglia cells (Supplementary Figure 2B).

### Ketamine Inhibits Cell Proliferation and Induces Cell Death in Oligodendrocyte Progenitor Cells

**FIGURE 3:**
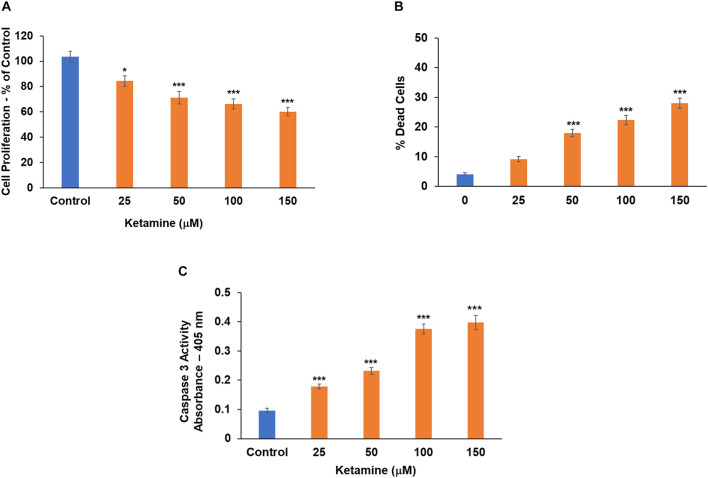
Ketamine inhibits cell proliferation and increases cell death in oligodendrocyte precursor cells. Human oligodendrocyte progenitor cells were treated with ketamine and analyzed 48 h later for cell proliferation **(A)** and cell death using the live/dead assay **(B)** and caspase 3 activity **(C)**. The results are the means ± SD of four independent test analyzed in triplicates. *P < 0.05 and ***P < 0.001.

Finally, we analyzed the effects of ketamine on the function of oligodendrocyte progenitor cells. Treatment with ketamine significantly decreased the proliferation of these progenitor cells (Figure 3A) and induced cell death in these cells in a dose-dependent fashion as determined by the live/dead assay (Figure 3B) and caspase 3 activity (Figure 3C). Thus, in contrast to microglia and astrocytes, neurons and oligodendrocyte progenitor cells exhibited an increased sensitivity to the neurotoxic effects of ketamine.

### Ketamine Differentially Modulates the BDNF Pathway in Human Neural Cells

Brain-derived neurotrophic factor (BDNF) plays important roles in neuronal growth and development and has been implicated as a mediator of GA neurotoxic effects (Lu et al., 2006; Ozer et al., 2015; Cavalleri et al., 2018; Fleitas et al., 2018). We focused on five main components of the BDNF pathway: mature BDNF, pro-BDNF, the lncRNA BDNF-AS and the expression of the BDNF receptors, p75 and TrkB.

**FIGURE 4:**
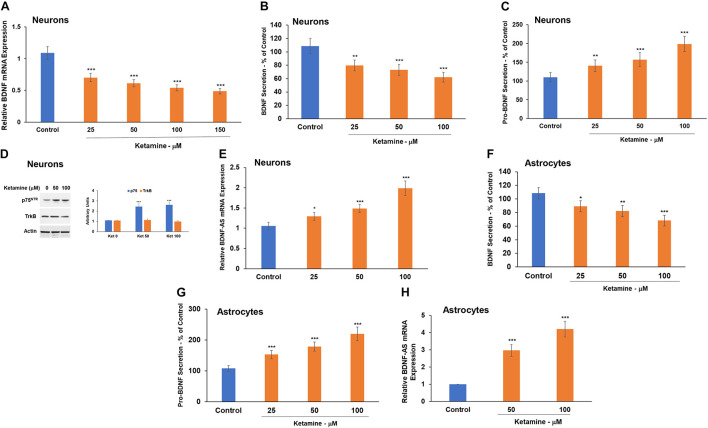
Ketamine regulates the BDNF pathway in neurons and astrocytes. Human neurons were treated with ketamine and the expression of BDNF mRNA was determined using RT-PCR **(A)** and BDNF secretion by ELISA **(B).** Pro-BDNF secretion was analyzed using ELISA **(C)** and the expression of p75^NTR^ and TrkB was analyzed using Western blot analysis **(D).** The effect of ketamine on BDNF **(F)** and pro-BDNF **(G)** secretion in astrocytes was analyzed using ELISA. The expression of the lncRNA BDNF-AS was analyzed using RT-PCR in both neurons **(E)** and astrocytes **(H).** The results are the means ± SD of three independent experiments analyzed in quadruplets or are a representative of three independent experiments **(D)**. *P < 0.05, **P < 0.01, and ***P < 0.001.

Treatment with ketamine decreased the expression (Figure 4A) and secretion (Figure 4B) of BDNF from cultured neurons. This effect was already observed at a concentration of 25 μM. In contrast, ketamine increased the secretion of pro-BDNF, as measured by ELISA (Figure 4C).

Brain-derived neurotrophic factor and pro-BDNF exert opposite effects *via* their preferential binding to TrkB and p75, respectively (Zheng et al., 2016; Fleitas et al., 2018). Therefore, an increased pro-BDNF secretion may promote neuronal cell death *via* binding to p75. We then analyzed the expression of TrkB and p75 in the cultured neurons and found that ketamine induced a modest upregulation of p75 but had no significant effect on the expression of TrkB in these cells (Figure 4D).

Another important component of the BDNF pathway is the lncRNA BDNF-AS. This lncRNA is transcribed from the opposite strand of BDNF and acts as a negative regulator of BDNF expression (Pruunsild et al., 2007; Zhang et al., 2018). We found that ketamine induced upregulation of BDNF-AS expression (Figure 4E), an effect that can contribute to the decrease in BDNF expression in the ketamine-treated cells.

Ketamine-treated astrocytes also exhibited a decreased BDNF (Figure 4F), an increased pro-BDNF secretion (Figure 4G) and a large increase in BDNF-AS expression (Figure 4H).

### Ketamine Induces an Increased Cell Death in Co-cultured Neuronal and Glial Cells

**FIGURE 5:**
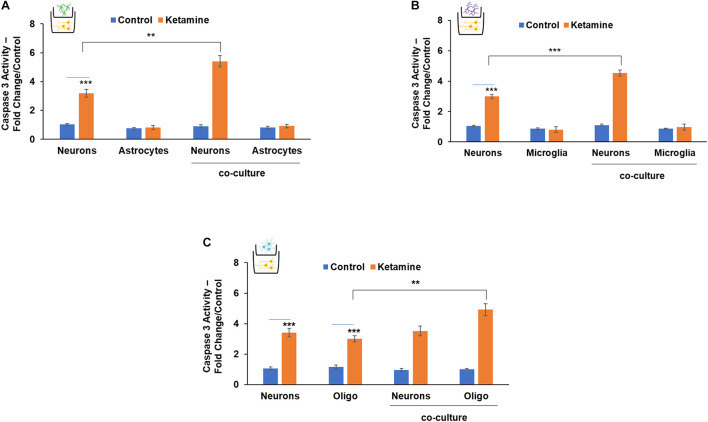
The role of neuron-glia interactions in the neurotoxic effects of ketamine. Neurons and astrocytes **(A),** neurons and microglia **(B)** or neurons and oligodendrocytes **(C)** were plated alone or co-cultured in transwell plates with a 1-μm filter. The co-cultures were treated with ketamine and caspase 3 activity was determined for the cultured cells after 48 h. The results are the means ± SD of six independent experiments analyzed in triplicates. ***P < 0.001 (co-cultured neurons vs. neurons alone **(A,B)** or co-cultured oligodendrocytes vs. oligodendrocytes alone **(C).** **P < 0.01 and ***P < 0.001 (ketamine-treated cells vs. controls).

We then analyzed the effects of ketamine in co-cultured neurons and glial cells using transwell plates with a 1 μm filter that allows only the transfer of soluble factors. Treatment of co-cultured neurons and astrocytes with ketamine resulted in enhanced neuronal cell death compared to neurons cultured alone. In contrast, no significant effects were observed on the cell death of the co-cultured astrocytes (Figure 5A). Similar effects were observed in co-cultures of microglia and neurons. Thus, ketamine treatment increased cell death of co-cultured neurons but had no significant effect on the death of co-cultured microglia cells (Figure 5B). In contrast, ketamine treatment of co-cultured neurons and oligodendrocyte progenitor cells resulted in increased cell death of the oligodendrocytes, without a significant effect on neuronal cell death (Figure 5C).

These results clearly demonstrate the complex and important neuronal-glial cell interactions in the neurotoxic effects of ketamine.

### EVs Play a Role in the Neurotoxic Effect of Ketamine

Extracellular vesicles play a role in the intercellular communication of neurons and glial cells (Basso and Bonetto, 2016; Datta Chaudhuri et al., 2020). To delineate the roles of EVs in the neuron-glial cell interactions in ketamine-treated cells, we isolated EVs from ketamine-treated astrocytes and examined their effects on neurons. The isolated EVs were analyzed by NTA for size distribution and concentration (Supplementary Figure 3A), for the expression of CD63 and CD81 using Western blot analysis (Supplementary Figure 3B), and for their amount using CD63 (Supplementary Figure 3C) and CD81 (Supplementary Figure 3D) ELISA. Ketamine treatment increased the secretion of both CD81 and CD63 + EVs by the treated astrocytes (Supplementary Figures 3C,D).

**FIGURE 6:**
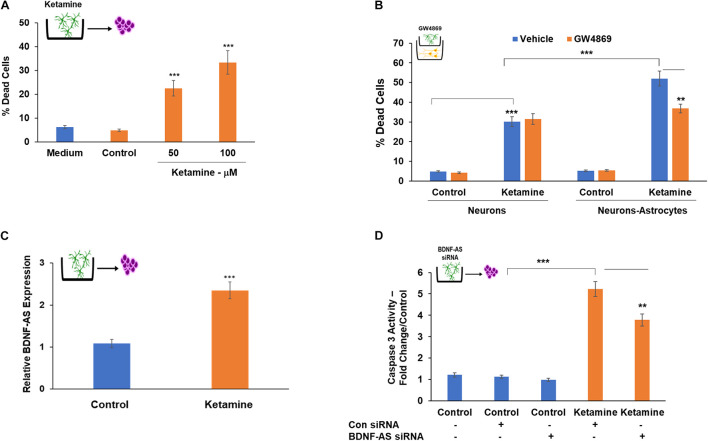
Astrocyte-secreted EVs mediate the increased neurotoxic effects of ketamine in neuron-astrocyte co-cultures. Cultured neurons were treated with EVs isolated from control or ketamine-treated astrocytes. Percent of dead cells was determined after 48 h **(A).** To further analyze the role of EVs secreted from astrocytes, these cells were pre-treated with GW4869 (20 μM) prior to ketamine treatment. The treated astrocytes were then co-cultured with neurons and treated with ketamine (50 μM). Neuronal cells death was analyzed after 48 h **(B)**. A similar treatment of GW4869 was performed in neuronal cells cultured alone **(B)**. The expression of BDNF-AS in EVs isolated from control and ketamine-treated astrocytes was analyzed by RT-PCR **(C).** EVs isolated from astrocytes that were silenced for BDNF-AS and then treated with ketamine were added to neuronal cultures. Cell death was determined after 48 h **(D)**. The results are the means ± SD of six independent experiments analyzed in quadruplets. **P < 0.01 and ***P < 0.001.

Extracellular vesicles isolated from astrocytes treated with ketamine for 24 h were added to cultured neurons for 48 h. As presented in Figure 6A, EVs isolated from ketamine-treated astrocytes induced neuronal cell death, whereas neurons treated with EVs from untreated cells (control) did not exhibit a significant increase in cell death, similar to that of untreated neurons (medium).

To further analyze the effects of EVs in the neurotoxic effects of co-cultured astrocytes, we employed the membrane neutral sphingomyelinase (nSMase) inhibitor, GW4869 which has been shown to reduce the secretion of EVs by blocking the ceramide-dependent budding of intraluminal vesicles (ILV) into the lumen of MVBs (Trajkovic et al., 2008). Astrocytes were treated with GW4869 (20 μM) prior to ketamine treatment of these cells in co-culture with neurons. We demonstrated that this treatment decreased EV secretion using CD63 ELISA (Supplementary Figure 3D). We found that GW4869 treatment partially abrogated the increased neurotoxic effect of astrocytes (Figure 6B) in the co-culture setting, whereas no significant effect of GW4869 on neuronal cell death was observed when these cells were cultured alone (Figure 6B). Altogether, these results indicate that EVs secreted from ketamine-treated astrocytes play at least a partial role in the increased neurotoxic response of co-cultured neurons.

Extracellular vesicles secreted from ketamine-treated astrocytes expressed higher levels of BDNF-AS compared to untreated cells (Figure 6C). To analyze the role of BDNF-AS in the neurotoxic effects of EVs isolated from ketamine-treated astrocyte, we silenced the expression of BDNF-AS in these cells. The cells were then treated with ketamine and EV were isolated. We found that the expression of BDNF-AS was significantly decreased in the EVs isolated from the silenced cells (Supplementary Figure 3F). EVs from ketamine-treated cells silenced for BDNF-AS exerted a significantly smaller increase in neuronal cell death compared to EVs isolated from cells transfected with a control siRNA (Figure 6D). These results indicate that the delivery of BDNF-AS by EVs secreted by ketamine-treated astrocyte plays a role in the neurotoxic effects of these EVs.

## Discussion

General anesthetics have been reported to induce neurotoxic effects in the developing brain that can be manifested in long-term cognitive impairments in rodent and primate models and in pediatric patients. However, the cellular and molecular mechanisms that are involved in GA effects and in particular the role of the neuron-glia interactions are not fully understood. Designing protective measures or alternative anesthetics require the understanding of the mechanisms that mediate GA neurotoxic effects. Although various studies aiming to delineate pathways that mediate these processes were reported in the last several years, most of them were performed in rodents (Jevtovic-Todorovic, 2016; Lin et al., 2017; Luo et al., 2020a).

Recent reports highlighted the differences in human and rodent neural cells and the need to employ reliable models to further understand the effects of GA in human cells and to supplement the animal model studies (Zhao and Bhattacharyya, 2018; Akter et al., 2020). Indeed, various studies reported the use of immortalized glial cells as reliable human models for analyzing specific therapeutic targets for various diseases including autism (Tsilioni et al., 2020), neuroinflammation (Timmerman et al., 2018; Chiavari et al., 2019) and for analyzing the crosstalk of glioma with glial cells (Henrik Heiland et al., 2019; Bier et al., 2020; Zeng et al., 2020). In these studies, we employed primary and immortalized human neural cells and analyzed the contribution of glial cells and neuron-glial interactions to ketamine’s neurotoxic effects and underlying molecular mechanisms. We demonstrated that the human neural cells employed in this study exhibited similar modes and dose-dependent responses to ketamine as previously reported studies in rodent and human cultured cells (Baker et al., 2016; Zhang et al., 2019; Kamp et al., 2020).

Astrocytes play major roles in various pathological conditions in the brain due to their key functions in hemostasis, maintenance, and glutamate uptake. Recently, astrocytes were demonstrated to undergo specific modes of activation depending on the pathological conditions in the brain (Liddelow and Barres, 2017; Hinkle et al., 2019; Koller and Chakrabarty, 2020). A1 astrocytes are induced by pro-inflammatory microglia, express specific markers such as C3 and are associated with various pathological conditions including neurodegenerative diseases and aging (Liddelow et al., 2017; Clark et al., 2019; Li et al., 2020). In contract, A2 astrocytes are induced by different stimuli like hypoxic conditions and exert neuroprotective effects (Li et al., 2019). It is now understood that the expression of specific genes and markers that is associated with each activation state appear to be pathology and brain region-dependent and multiple intermediate activation states are being identified (Guttenplan et al., 2020; Escartin et al., 2021). We report for the first time that ketamine increased the expression of the A1 activation marker, C3 and decreased the expression of the A2 marker S100A10. Additional studies are needed to further characterize the activation of astrocytes by ketamine and RNA sequencing studies are currently being performed to further define the specific gene and non-coding RNAs expressed in these cells.

Ketamine also downregulated the expression of the glutamate transporter, EAAT2 in astrocytes. Astrocytes represent the main cells in the brain that remove glutamate from the synaptic cleft *via* membranal glutamate transporters (Mahmoud et al., 2019). Because of this important role, changes in astrocyte activation and function can result in glutamate-related pathological conditions. The effect of ketamine on the expression of EAAT2 in human astrocytes has not been previously described. However, there are studies demonstrating that ketamine significantly increases synaptic glutamate release in cortex and striatum while decreasing the expression of membrane EAAT2 as part of the mechanism of the psychogenic effect of ketamine (Lisek et al., 2017). In addition, ketamine in clinical concentrations, inhibits glutaminergic transmission from astrocytes to neurons (Zhang et al., 2019).

Ketamine also increased the expression of M1-proinflammatory markers in microglial cells. Microglia play major roles during brain development by providing neuronal neurotrophic support and phagocytosis of apoptotic cells during this process (Al-Onaizi et al., 2020). In the mature brain microglia regulate synaptic organization and neuronal excitability, myelin turnover, inflammatory responses and injury and repair mechanisms (Wake and Fields, 2011). Similar to macrophages, microglia can differentiate to different subtypes with relative characteristics of M1/M2 phenotypes consisting of a broad array of activation profiles (Nakagawa and Chiba, 2014; Tang and Le, 2016). Various neuroinflammatory and neurological diseases are characterized by a relative increase in M1 microglia-related factors such as TNF-α and IL-1β (Zhang et al., 2017; Huang et al., 2019). The relative increase in M1 phenotypes by ketamine is in line with its effects on the A1 activation of astrocytes. Altogether, ketamine may upregulate neuroinflammation and neurotoxicity by promoting microglia and astrocyte activation.

Ketamine induces demyelination in the developing brain (Patel and Sun, 2009). However, its effects on human oligodendrocytes have not been reported. We found that oligodendrocyte progenitor cells, exhibited an increased cell death in response to ketamine, similar to the effect observed in neurons. Therefore, our results may provide a cellular basis for the demyelinating effects of ketamine.

Ketamine and other GAs induce neurotoxicity and long-term cognitive impairments *via* various molecular mechanisms including changes in apoptosis-related genes, secretion of cytokines and neurotrophic factors (Zuo et al., 2016). We focused on the effect of ketamine on the BDNF pathway and found that ketamine decreased BDNF secretion from both neurons and astrocytes, while increasing the secretion of pro-BDNF in these cells. Moreover, ketamine also increased the expression of the p75^NTR^ in the treated neurons. Changes in the levels of BDNF/pro-BDNF in the CNS have been reported in various neurological conditions and as potential mechanisms of neuronal cell death (Luo et al., 2019, 2020b). BDNF exerts neurotrophic effects *via* binding to both p75^NTR^ and TrkB, while pro-BDNF binds only to the p75^NTR^ receptor and activates a variety of processes involving synapse structure and function, and cell death (Teng et al., 2005). The increased p75^NTR^ and pro-BDNF expression are associated with a variety of pathological processes such as memory impairment (Buhusi et al., 2017), increased susceptibility to epileptic seizures (Riffault et al., 2018) and neuronal apoptosis (Teng et al., 2005).

Ketamine impacted the levels of BDNF also by increasing the expression of the lncRNA BDNF-AS in neurons and to a larger degree in astrocytes. This lncRNA is transcribed from the opposite strand of BDNF and acts as a negative regulator of BDNF expression (Pruunsild et al., 2007; Lipovich et al., 2012). Indeed, BDNF-AS has been associated with multiple CNS injuries (Zhang and Wang, 2019), neonatal hypoxia/ischemia-induced brain injury (Qiao et al., 2020), Parkinson’s disease (Palasz et al., 2020) and neuronal aging (Pereira Fernandes et al., 2018). Altogether, the decreased expression of BDNF and the increased expression of BDNF-AS, p75^NTR^ and pro-BDNF in the ketamine-treated cells may represent one of the major pathways of the neurotoxic effects of ketamine.

There is now powerful evidence for non-cell autonomous mechanisms in which neurotoxicity is strongly affected by changes in both neuronal and non-neuronal cells in almost every pathological condition in the brain (Lobsiger and Cleveland, 2007; Ilieva et al., 2009). Although there are some studies demonstrating a role of astrocytes (Zhang et al., 2019; Zhou et al., 2019) and microglia (Baud and Saint-Faust, 2019) in GA effects, the non-cell autonomous effects of different glial cells were not consistently studied and identified. Our co-culture studies indicate that ketamine treatment of astrocytes and microglia increased co-cultured neuronal cell death, while neuronal treatment increased the apoptosis of co-cultured oligodendrocytes, indicating a major contribution of the neuron-glia effects to the neurotoxic effect of ketamine. One potential mechanism for the ketamine neurotoxicity in co-cultured cells is the increased pro-BDNF that is associated with the increased cell death in neurons and oligodendrocytes *via* activation of the p75 pathway.

Another potential and novel mechanism of ketamine effect is mediated by EVs. We showed for the first time that ketamine increased the amount of EVs secreted by astrocytes and that these EVs induced cells death in neuronal cells. In addition, inhibition of EV secretion in astrocytes abrogated the increased neuronal cell death observed in co-cultures. Indeed, EVs have been implicated as important components of neuron-glia intercellular communication (Yin et al., 2020) and as mediators of various pathological conditions in the brain (Budnik et al., 2016; Hill, 2019). We also demonstrated that ketamine treatment of astrocytes increased the expression of BDNF-AS in EVs secreted from these cells. We further found that the increased secretion of this lncRNA was associated with the cytotoxic effects of the EVs on neuronal cells. Current ongoing studies aim to analyze the cargo of the EVs secreted by the ketamine-treated astrocytes and additional neurotoxic factors involved in their effects.

**FIGURE 7:**
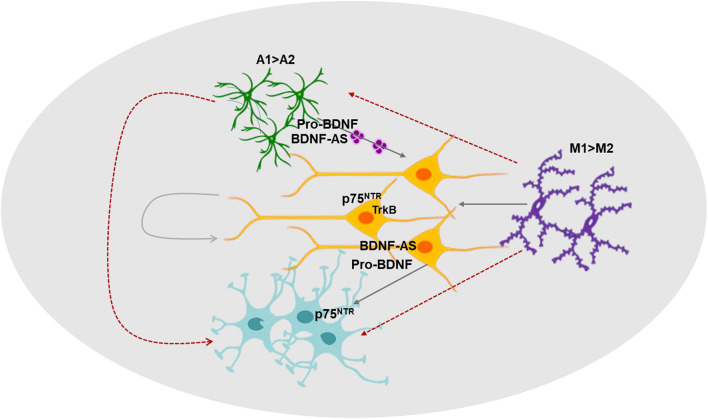
A diagram summarizing the roles of neuron-glia interactions, the BDNF pathway and extracellular vesicles in ketamine’s effects. The effects of ketamine on various components of the BDNF pathway, the interactions of neuron and glial cells and the role of EVs as mediators of these interactions are depicted in this diagram.

In summary, we demonstrated multicellular ketamine effects on human neural cells, an increased expression of A1 astrocytes and M1 microglia markers and that ketamine induces neurotoxic effects by both cell autonomous and non-cell autonomous mechanisms. Moreover, we identified important and integrated roles of multiple components of the BDNF pathway in ketamine neurotoxic effects. Finally, we demonstrated for the first time that ketamine treatment affects the secretion, cargo and function of astrocyte-derived EVs. The secreted EVs increase neuronal cell death and act as important mediators of neuron-astrocyte interactions in the neurotoxic action of ketamine. These findings may have a broad implication in the short- and long-term effect of this GA (Figure 7).

## Data Availability Statement

The authors acknowledge that the data presented in this study must be deposited and made publicly available in an acceptable repository, prior to publication. Frontiers cannot accept a manuscript that does not adhere to our open data policies.

## Author Contributions

CB and DP: concept and design. CB and SC: development of methodology and data acquisition. CB, DP, AB, and VJ-T: data analysis and interpretation. CB, DP, AB, ML, and SK: writing and review of the manuscript. All authors contributed to the article and approved the submitted version.

## Conflict of Interest

AB was employed by Precise Cell Ltd. The remaining authors declare that the research was conducted in the absence of any commercial or financial relationships that could be construed as a potential conflict of interest.

## Publisher’s Note

All claims expressed in this article are solely those of the authors and do not necessarily represent those of their affiliated organizations, or those of the publisher, the editors and the reviewers. Any product that may be evaluated in this article, or claim that may be made by its manufacturer, is not guaranteed or endorsed by the publisher.

## Abbreviations

BDNF: brain-derived neurotrophic factor
CNS: central nervous system
EVs: extracellular vesicles
GABA_*A*_R: gamma-amino-butyric acid type-A receptors
GAs: general anesthetics
NMDAR: *N*-methyl -D-aspartate receptors.
