# Supplementary figures and images for: Neuron-Glia Crosstalk Plays a Major Role in the Neurotoxic Effects of Ketamine via Extracellular Vesicles
Source: Front Cell Dev Biol. 2021 Sep 16;9:691648. doi: 10.3389/fcell.2021.691648 (PMC8481868; doi:10.3389/fcell.2021.691648)

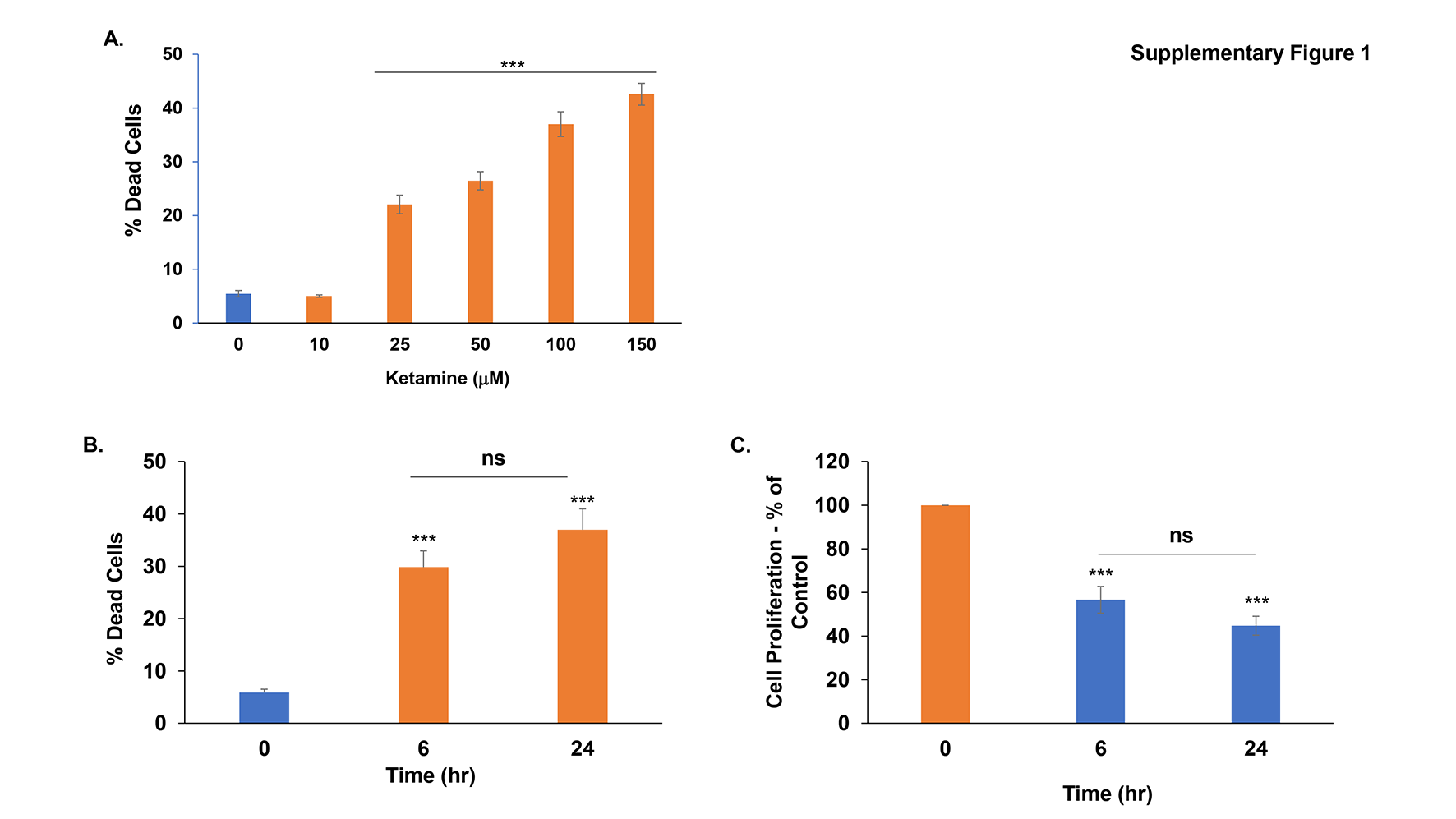

Supplement: Supplementary file 1 [file Image_1.tif]

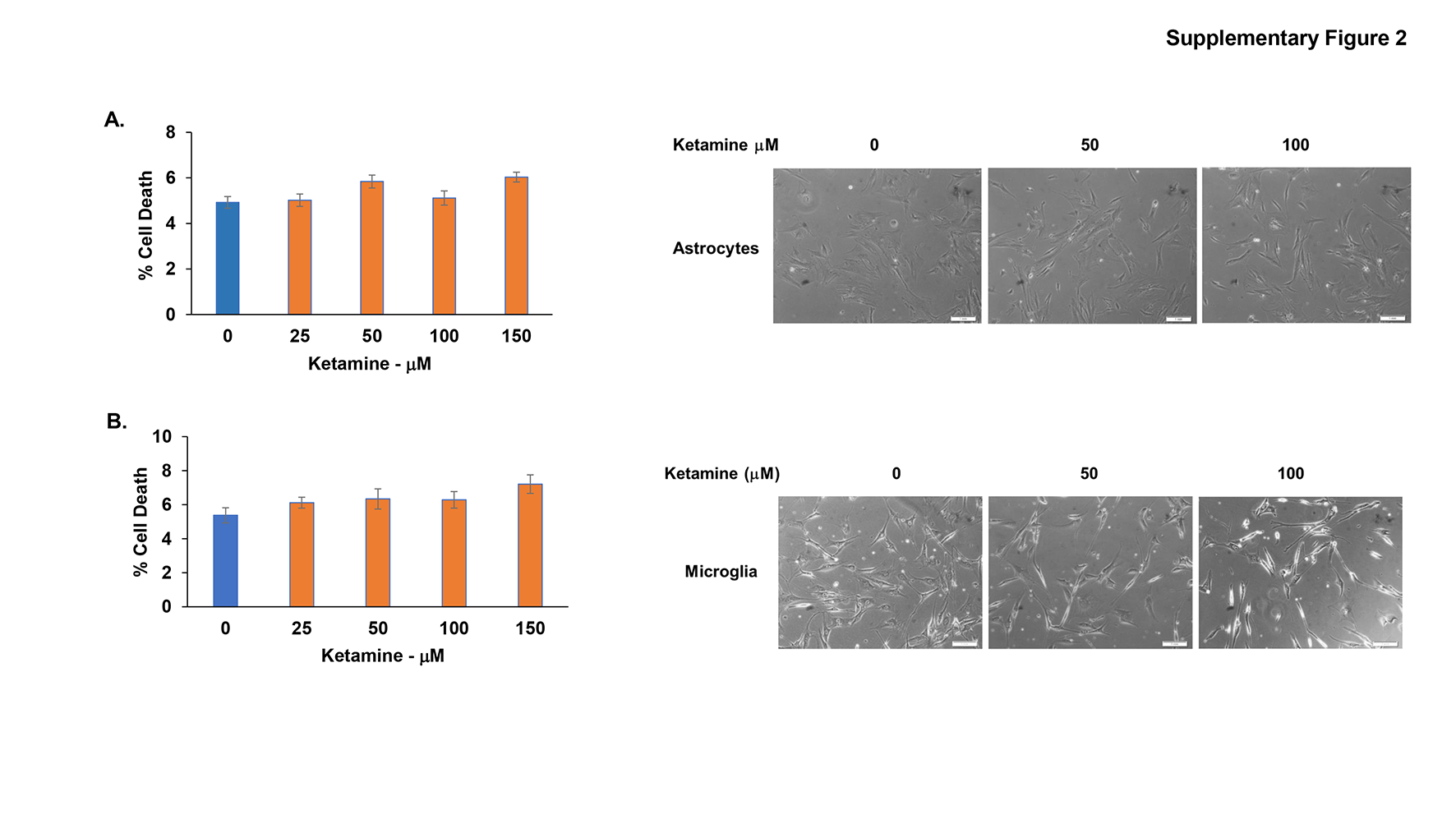

Supplement: Supplementary file 2 [file Image_2.tif]

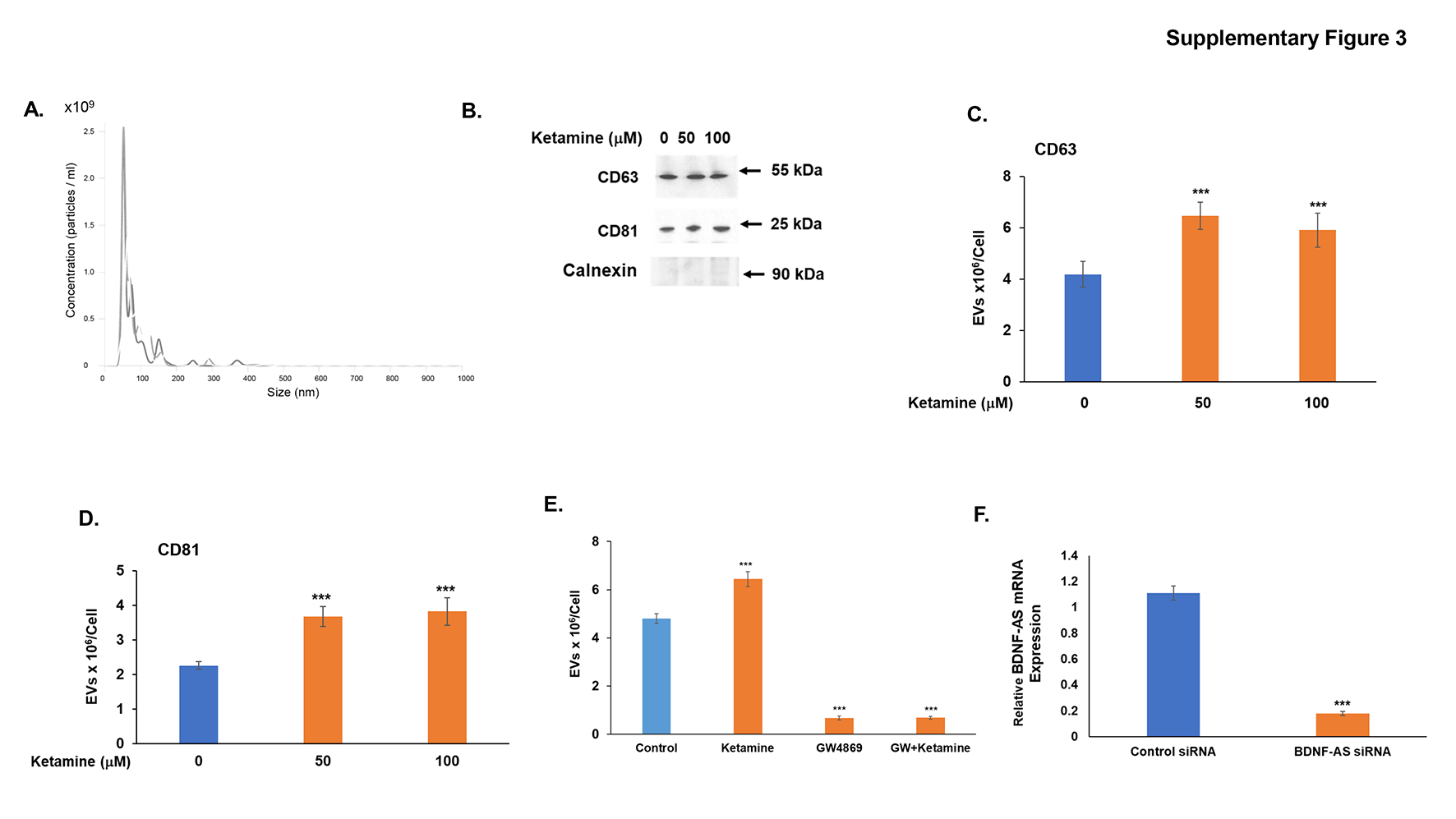

Supplement: Supplementary file 3 [file Image_3.tif]
