# Supplementary material for: Neuron-Glia Crosstalk Plays a Major Role in the Neurotoxic Effects of Ketamine via Extracellular Vesicles
Source: Front Cell Dev Biol. 2021 Sep 16;9:691648. doi: 10.3389/fcell.2021.691648 (PMC8481868; doi:10.3389/fcell.2021.691648)
Supplement: Supplementary file 4 [file Data_Sheet_1.docx]

**Supplementary figure legends**

**Figure S1. Effects of ketamine on cell death and proliferation of neurons and oligodendrocyte progenitors**. Human neurons (A, B) and human oligodendrocyte progenitors (C) were treated with different concentrations of ketamine (A) or with 100 μM for 6 and 24 hr. Cell death (A,B) and proliferation (C) were determined 24 hr thereafter.

The results are the means ± SD of a representative out of three independent experiments analyzed in quadruplet. ***P<0.001 compared to untreated cells.

**Figure S2. Effects of ketamine on cell death and morphology and astrocytes and microglial cells.** Human astrocytes and microglial cells were treated with different concentrations of ketamine for 72 he. Cell death and morphology was determined for astrocytes (A) and Microglia cells(B). The results are the means ± SD of a representative experiment of three separate tests analyzed in quadruplet.

**Figure S3. Marker expression and quantification of ketamine-treated EVs.** The size distribution of EVs isolated from astrocytes was determined by NTA (mode = 82 ± 5 nm) (A). Astrocytes were treated with ketamine (50 and 100 μM) for 6 hr and EVs were collected after 24 hr. The expression of the EV markers, CD63 CD81 and the ER protein (a cell marker), calnexin, was determined by Western blot analysis (B). The amount of the secreted EVs was measured using CD63 (C) and CD81 (D) ELISA kits (B). Astrocytes were treated with GW649 ( 20 μM) and the amount of secreted EVs was determined using CD63 ELISA (E). Astrocytes were transfected with control or BDNF-AS siRNAs and treated with ketamine. EVs were isolated and the expression of BDNF-AS was determined using RT-PCR (F). The results are the means ± SD of three independent experiments or the means ± SD of a representative experiment of three separate tests analyzed in quadruplet (F) or the . ***P<0.001 ***P<0.001
