# Supplementary material for: Neuron-Glia Crosstalk Plays a Major Role in the Neurotoxic Effects of Ketamine via Extracellular Vesicles
Source: Front Cell Dev Biol. 2021 Sep 16;9:691648. doi: 10.3389/fcell.2021.691648 (PMC8481868; doi:10.3389/fcell.2021.691648)
Supplement: Supplementary file 5 [file Table_1.docx]

**Table S1**

**Sequences of primers used for RT-PCR**

| **S100A10 S:** CAAATGGAACACGCCATGGAA **AS**:AGCCCACTTTGCCATCTCTAC |
| --- |
| **C3** **S**: AAAGAGGACATCCCACCTGC **AS**: GGGGTCACAATGAGGTGCTT |
| **BDNF S:** CCCCCATGAAAGAAGCAAAC **AS:** CGTGTTCGAAAGTGTCAGCC |
| **BDNF-AS** **S**: CCGTGAGAAGATCTCATTGGG **AS**: GGGTCACAAGTCACGTAGCA |
| **IL-13** **S**: CGAGAAGACCCAGAGGATG **AS**: GTCTCGGACATGCAAGCTG |
| **IL-1α** **S**: CCAAGATGAAGACCAACCA **AS**: GCCAAGCACACCCAGTAGT |
| **EAAT2** **S**: CGCTGTTGTCTCTCTGTTGA **AS**:ATCTCCCATCTTCCCCATAG |
| **CD68**  **S**: ACAATGTGTCCTTCCCCCAC **AS**: CCGATGATGAGAGGCAGCAA |
| **CD206**  **S**: CATCAGGGTGCAAGGAAGG **AS**: GTCCAGGCACTGAAAGTGGA |
| **S12 S**:TGCTGGAGGTGTAATGGACG **AS**: CAAGCACACAAAGATGGGCT |
